# Fabrication of BaTiO_3_-Loaded Graphene Nanosheets-Based Polyarylene Ether Nitrile Nanocomposites with Enhanced Dielectric and Crystallization Properties

**DOI:** 10.3390/nano9121667

**Published:** 2019-11-22

**Authors:** Shuning Liu, Chenchen Liu, Yong You, Yajie Wang, Renbo Wei, Xiaobo Liu

**Affiliations:** Research Branch of Advanced Functional Materials, School of Materials and Energy, University of Electronic Science and Technology of China, Chengdu 611731, China; liushuningg@126.com (S.L.); liuchenchen@std.uestc.edu.cn (C.L.); yourkeaib@163.com (Y.Y.); m18380127314@163.com (Y.W.)

**Keywords:** nanoparticles, nanocomposites, electrostatic attraction, dielectric properties

## Abstract

In this paper, barium titanate@zinc phthalocyanine (BT@ZnPc) and graphene oxide (GO) hybrids (BT@ZnPc-GO) connected by calcium ions are prepared by electrostatic adsorption, and then introduced into polyarylene ether nitrile (PEN) to obtain composites with enhanced dielectric and crystallization properties. Scanning electron microscopy (SEM), transmission electron microscopy (TEM), X-ray photoelectron spectroscopy (XPS), X-ray diffraction (XRD) and Fourier transform infrared spectroscopy (FTIR) results confirm the successful fabrication of the BT@ZnPc-GO. BT@ZnPc-GO and PEN composites (BT@ZnPc-GO/PENs) are obtained through the solution-casting method. BT@ZnPc-GO demonstrates well compatibility with PEN due to its unique structure and the organic layer of ZnPc at the periphery of BT. On the other hand, BT and GO contribute a high dielectric constant of the composites obtained. In addition, the BT@ZnPc-GO can be used as a nucleating agent to promote the crystallization of the nanocomposites. As a result, The BT@ZnPc-GO/PEN exhibits a dielectric constant of 6.4 at 1 kHz and crystallinity of 21.03% after being isothermally treated at 280 °C for 2 h at the GO content of 0.75 wt %. All these results indicate that the hybrid nanofiller BT@ZnPc-GO can be an effective additive for preparing high-performance PEN-based nanocomposites.

## 1. Introduction

To fulfill the increasing requirements of the electronics industry, high-temperature-resistant, integrated, flexible and high-k dielectric materials play an increasingly important role in capacitors, electromagnetic interference (EMI) shielding materials and microelectronic components fields [1,2,3]. Recently, polymer-based nanocomposites, combing the excellent properties of the polymer matrix and the fillers, have been widely used by the scientific community as high-energy-density films in the electronics industry due to their excellent properties such as their lightness, flexibility, high dielectric constant and excellent thermal stability [4,5,6].

As an inorganic filler in a composite material, the ferroelectric ceramics, such as barium titanate (BaTiO_3_), strontiumtitanate (SrTiO_3_), calcium titanate (CaTiO_3_) are generally used to increase the dielectric constant of the polymer [7,8] owing to their outstanding high dielectric distance and low dielectric loss. Unfortunately, most inorganic ceramic nanofillers are extremely easy to agglomerate in the polymer matrix due to their large specific surface area, high surface energy and weak interface interaction, which will seriously affect the mechanical properties and compatibility of the polymer-based nanocomposites [9]. Therefore, typically modifications of the ferroelectric ceramic nanofillers are always needed to increase the compatibility with the polymer matrix, thereby reducing the impact on the dielectric properties [10]. Furthermore, the addition of conductive particles such as metal particles, graphene, carbon fibers or carbon nanotubes is very effective for increasing the dielectric constant of the polymer system with a low addition amount [11,12]. Graphene oxide (GO), a typical two-dimensional layer structure, can be used as a carrier for the deposition of inorganic nanoparticles such as Fe_3_O_4_, BaTiO_3_ and TiO_2_ on its surface to obtain new hybrids, showing excellent electrical and magnetic properties [13]. As another important part of the nanocomposite materials, the polymer matrix will be directly related to the properties of the nanocomposites. Polyarylene ether nitrile (PEN), a kind of high-performance thermoplastic aromatic polymer, shows high heat resistance, flame retardancy, radiation resistance, excellent mechanical strength, outstanding flexibility and formability [14,15,16,17]. In addition, PEN still has significant advantages over other thermoplastic resins such as thermoplastic polyimide (TPI) [18] and polyetheretherketone (PEEK) [19], including low processing temperature (~280 °C), high temperature resistance (>450 °C) and short molding time (0.5~1 h), and these advantages are beneficial for processing PEN into various forms to meet the dielectric applications.

In this work, barium titanate@zinc phthalocyanine/graphene oxide (BT@ZnPc-GO) nanoparticles with different GO contents were firstly prepared by electrostatic adsorption between BT@ZnPc and GO nanoparticles with the action of calcium ion, and the obtained nanoparticles are characterized in detail. In addition, the BT@ZnPc-GO nanoparticles were regarded as the hybrid nanofillers to incorporate into the PEN matrix for preparing the BT@ZnPc-GO/PEN nanocomposites. The corresponding thermal and dielectric properties of the nanocomposites were further systematically studied.

## 2. Materials and Methods

### 2.1. Materials

**Scheme 1:**
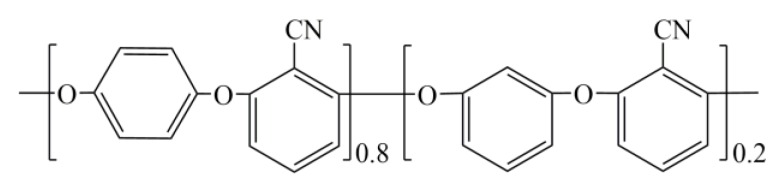
Structure of polyarylene ether nitrile (PEN).

BT@ZnPc was prepared according to the previous work, BT@ZnPc was prepared by mixed for sonication in *N*-methyl-2-pyrrolodone (NMP) for 3 h and the filtered mixture was treated at 200 °C for 4 h [20]. Calcium chloride (CaCl_2_) and potassium carbonate (K_2_CO_3_) were purchased from Tianjin BODI chemicals, Tianjin, China. GO was synthesized from graphite powder according to an improved Hummer’s method as reported [21]. PEN was synthesized by 2, 6-dichlorobenzonitrile and biphenol (hydroquinone (HQ)/resorcinol (RS) = 8/2 by mol) according to previous reports in our laboratory, and the corresponding structure is shown in Scheme 1.

### 2.2. Preparation of Barium Titanate@Zinc Phthalocyanine and Graphene Oxide Hybrid (BT@ZnPc-GO)

**Scheme 2:**
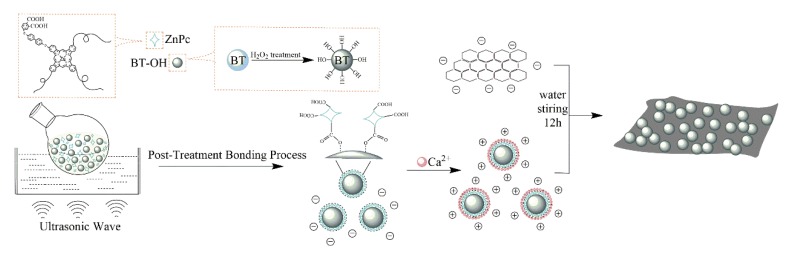
Preparation route of barium titanate@zinc phthalocyanine and graphene oxide hybrid (BT@ZnPc-GO).

2 g CaCl_2_ was firstly dissolved in 200 mL deionized water, and then the 100 mg BT@ZnPc nanoparticles were added in the CaCl_2_ solution with mechanical stirring and ultrasonic wave for 30 min. The obtained solution was filtered and washed by deionized water 3 times to remove excess CaCl_2_ until the filtrate droplets K_2_CO_3_ were added an no turbidity appears, and were washed with deionized water several times. Meanwhile, an appropriate amount of GO was dispersed in 10 mL deionized water by using ultrasonic waves for 30 min and then added into the obtained BT@ZnPc/Ca^2+^ solution, and the mixture were continuous stirred for 12 h. After filtration, the solid product was dried in a vacuum oven at 80 °C for 12 h to obtain the BT@ZnPc-GO nanoparticles. The specimens of GO of 5 and 10 mg are named BT@ZnPc-GO-1 and BT@ZnPc-GO-2, respectively. The corresponding preparation diagram is shown in Scheme 2.

### 2.3. Preparation of BT@ZnPc-GO/Polyarylene Ether Nitrile (PEN) Nanocomposites

The BT@ZnPc-GO/PEN composite films with 0, 1, 3, 5, 10 and 15 wt % BT@ZnPc-GO nanoparticles were prepared by solution casting method. Firstly, a certain amount of BT@ZnPc-GO nanoparticles were uniformly dispersed in NMP solvent with the mechanical stirring and ultrasonic wave for 30 min. Afterwards, 1 g PEN was added in the dispersion and stirred continuously for about 20 min at 80 °C until it was completely dissolved. After that, the mixture obtained was casted on a horizontal glass and dehydrated under a preset program based on the previous method [20]. After being cooled down to room temperature, BT@ZnPc-GO/PEN nanocomposite films were successfully fabricated.

### 2.4. Characterization

Scanning electron microscopy (SEM) was applied using a JEOL JSM-5900LV (Tokyo, Japan) and transmission electron microscopy (TEM) was applied with a ZEISS Libra 200 FE (Oberkochen, Germany). X-ray diffraction (XRD) was obtained using an X-ray diffractometer (Karlsruhe, Germany) under Cu Kα radiation. Differential scanning calorimetry (DSC) analysis was tested with the TA Instrumens DSC Q100 (NewCastle, DE, USA). Fourier transform infrared spectroscopy (FTIR) and X-ray photoelectron spectroscopy (XPS) were measured with a Shimadzu 8400S FTIR spectrophotometer and ESCA 2000 from VG Microtech (London, UK). Zeta potential was obtained by Malvern ZetasizerNano ZS90 (Taiwan, China) and the dielectric propreties were carried out using TH 2819A precision LCR meter (Changzhou, China).

## 3. Results and Discussion

In this work, barium titanate@zinc phthalocyanine/grapheneoxide (BT@ZnPc-GO) nanoparticles were prepared by electrostatic adsorption between BT@ZnPc and GO nanoparticles with the action of calcium ions. As can be seen from Scheme 2, BT@ZnPc was firstly prepared by an esterification reaction between the carboxyl group of ZnPc-COOH and the hydroxyl group of BT-OH which was treated with hydrogen peroxide. Secondly, the surface of the BT@ZnPc nanoparticles will present the positive charge after the addition of CaCl_2_. As different amounts of negatively charged GO/water solution were gradually dropped into the BT@ZnPc/Ca^2+^ solution, the BT@ZnPc-GO-1 and BT@ZnPc-GO-2 nanoparticles were successfully prepared by electrostatic attraction between the positively charged BT@ZnPc nanoparticles and the negatively charged GO in aqueous media [22].

**Figure 1:**
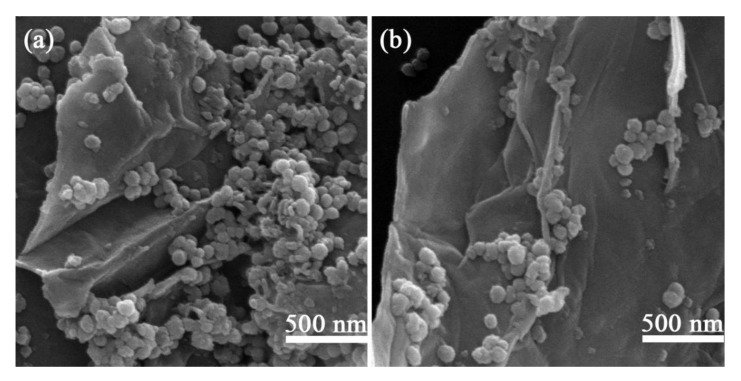
Scanning electron microscope (SEM) image of (**a**) BT@ZnPc-GO-1 and (**b**) BT@ZnPc-GO-2.

In order to observe the microstructure of the obtained nanoparticles intuitively, the BT@ZnPc-GO nanoparticles with different content of GO were characterized by SEM, which are shown in Figure 1. It is clearly that the all the BT@ZnPc nanoparticles were stick tightly to the surface of the GO nanosheets, indicating that a strong surface electrostatic adsorption force exists between the GO nanosheets and BT@ZnPc nanoparticles. In addition, with the increase of GO content, the average surface area for the nanoparticles to attach increased. Therefore, augmenting the amount of GO sheets will relatively reduce the number of BT@ZnPc nanoparticles on a GO sheet. Thus the size of a cluster of BT@ZnPc nanoparticles/GO sheet will be also reduced, which indicated that the GO nanosheets can be used as a surfactant to promote the uniform dispersion of the BT@ZnPc nanoparticles.

**Figure 2:**
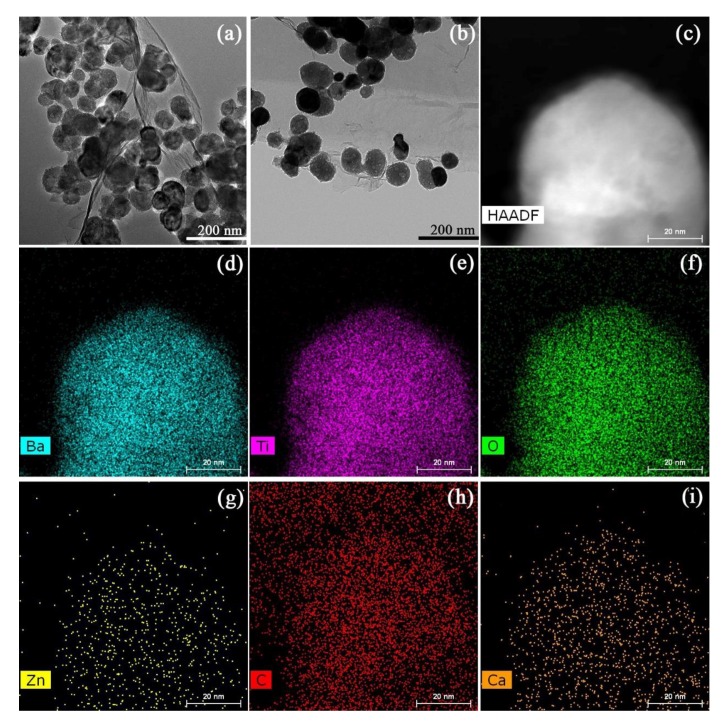
(**a**) Transmission electron microscope (TEM) image of BT@ZnPc-GO-1, (**b**) TEM image of BT@ZnPc-GO-2. (**c**) STEM images of BT@ZnPc-GO-1. EDS mapping images of (**d**) Ba, (**e**) Ti, (**f**) O, (**g**) Zn, (**h**) C and (**i**) Ca.

In addition, to further investigate the microstructure and the component of the nanoparticles, the TEM and the TEM energy dispersive spectroscopy (EDS) mapping images of BT@ZnPc-GO nanoparticles are shown in Figure 2. As can be seen in Figure 2a, the graphene oxide nanosheets are well coated by BT@ZnPc nanoparticles, which densely and evenly deposited on both sides of these sheets to form a sandwich-like composite structure. Moreover, it can clearly be seen that BT@ZnPc-GO-2 has more uniform distribution and the graphene oxide nanosheets were well coated by BT@ZnPc nanoparticles, which are shown in Figure 2b. The scanning transmission electron microscope (STEM) image and of BT@ZnPc-GO-1 EDS mapping images of Ba, Ti, O, Zn, C and Ca are exhibited in Figure 2c–i. It is obvious that the color distribution points of all different elements are evenly present in BT@ZnPc-GO-1, which indicated that the BT@ZnPc nanoparticles are homogeneously deposited on the graphene oxide sheets. Meanwhile, the EDS spectrum of calcium (Figure 2i) shows that calcium is almost exclusively present on the surface of the BT@ZnPc nanoparticles, indicating that calcium ions promote the electrostatic attraction between BT@ZnPc nanoparticles and GO nanosheet in this system.

**Figure 3:**
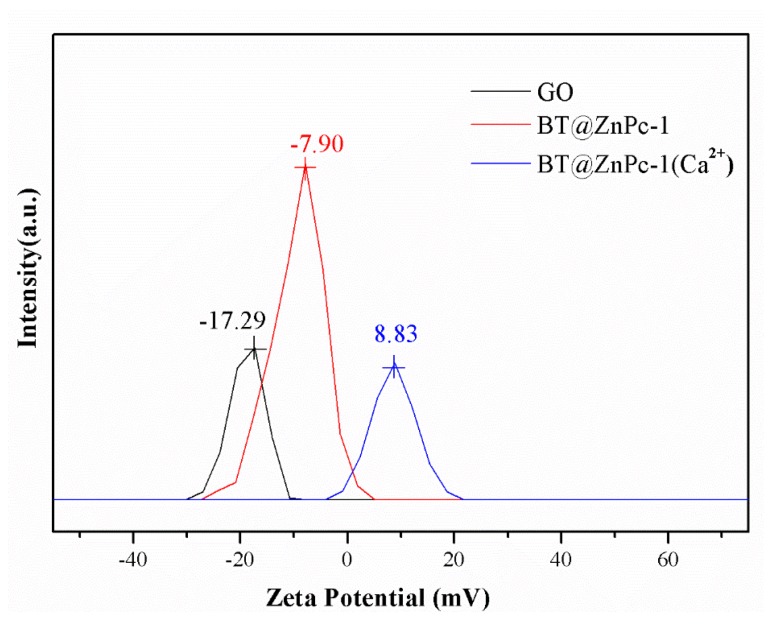
Intensity of light-scattering versus zeta potential (calculated based on applied voltage) in electrophoresis through suspension of charged particles at pH = 7.

Figure 3 shows the intensity of light scattering as a function of the zeta potential of GO, BT@ZnPc-1 and BT@ZnPc-1 after modified by Ca^2+^ particles at pH = 7. Zeta potential refers to the potential of the Shear Plane, which is related to the total charge carried by the particles in a particular medium and the peak of the zeta potential represents the type and intensity of charge present after dispersed in solution [23]. It can clearly be seen that the highest light scattering of GO is observed at the zeta potential of −17.29 mV, which is mainly owing to the ionization of oxygenated functional groups in a high polar solvent like water, and the pristine GO would assume slightly negative charge. Moreover, the highest light scattering of BT@ZnPc-1 is −7.90 mV, which is due to the fact that the zinc phthalocyanine in BT@ZnPc is carboxylated, and the –COOH groups on the surface of BT@ZnPc nanoparticles are partially hydrolyzed in an aqueous medium to form some –COO^–^, making the BT@ZnPc-1 electrostatically negatively charged. However, the modification of calcium ions on the surface of BT@ZnPc nanoparticles will provide a positively charged surface. Therefore, the positively charged BT@ZnPc/Ca^2+^ nanoparticles interact with negatively charged GO nanosheets to form electrostatically neutral particles.

**Figure 4:**
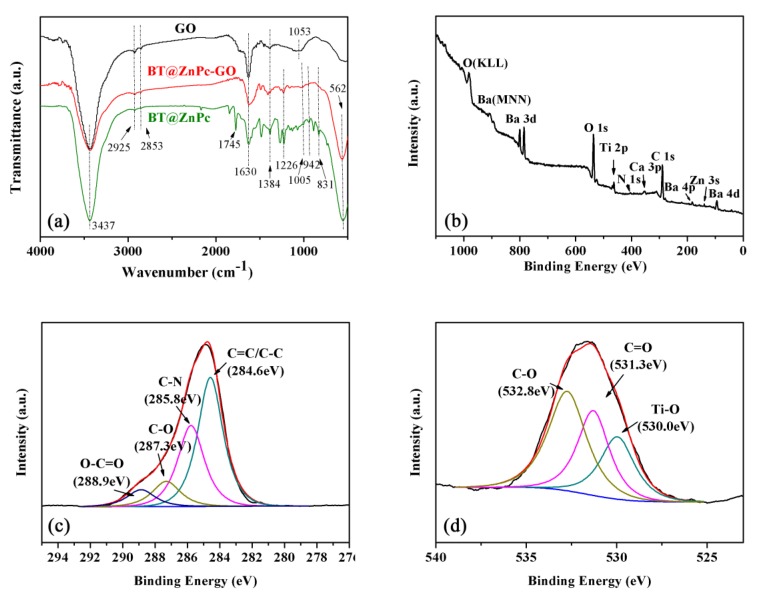
(**a**) Fourier transform infrared spectroscopy (FTIR) spectra of GO, BT@Znpc-GO-1 and BT@ZnPc; (**b**) X-ray photoelectron spectroscopy (XPS) spectrum of BT@ZnPc-GO-1; (**c**) XPS C1S spectra of BT@ZnPc-GO-1; (**d**) XPS O1S spectra of BT@ZnPc-GO-1.

Furthermore, the chemical structure of the prepared BT@ZnPc-GO nanomaterials is characterized by FTIR and XPS, which are shown in Figure 4. As can be seen from Figure 4a, the BT@ZnPc-GO and BT@ZnPc exhibit a same strong absorption at 562 cm^−1^, which belongs to Ti–O vibration. The three obvious absorption peaks at 1005, 942 and 831 cm^−1^ are belonging to the absorption of phthalocyanine [24]. The characteristic absorption peak appearing at 1630 and 1384 cm^−1^ of all samples belongs to the carboxyl group, and the absorption peak at 2853 and 2925 cm^−1^ belongs to C–H. Besides, the absorption peak at 1745 cm^−1^ in the curve of BT@ZnPc presents the formation of the ester bond in BT@ZnPc, indicating a reaction has occurred between the carboxyl from the ZnPc-COOH and the hydroxyl on the peripheral of BT-OH [20]. In addition, Figure 4b-d show the XPS spectrums of the prepared BT@ZnPc-GO nanoparticles. As shown in Figure 4b, the C 1s, O 1s, N 1s, Ti 2p, Zn 3s and Ba 3d, 4d and 4p can be observed from the full scanned survey spectrum of BT@ZnPc-GO, which indicates the presence of BT@ZnPc and GO. Moreover, the C1s spectrum of BT@ZnPc-GO can be quantitatively differentiated into four different carbon species located at 284.6, 285.8, 287.3 and 288.9 eV [25], which belong to the chemical bonds of the carbon-carbon bond (C = C/C–C), carbon-nitrogen bond (C–N) on phthalocyanine ring, carbon-oxygen bond (C–O) and carboxyl bond (O–C = O) respectively (Figure 4c). The O1s spectrum of BT@ZnPc shows the titanium oxide bond (Ti–O) at 530.0 eV, carbon–oxygen double bond (C = O) at 531.3 eV and carbon-oxygen single bond (C–O) at 532.8 eV. Therefore, according to the characterizations of FTIR and XPS, the BT@ZnPc-GO nanoparticles are successfully prepared.

**Figure 5:**
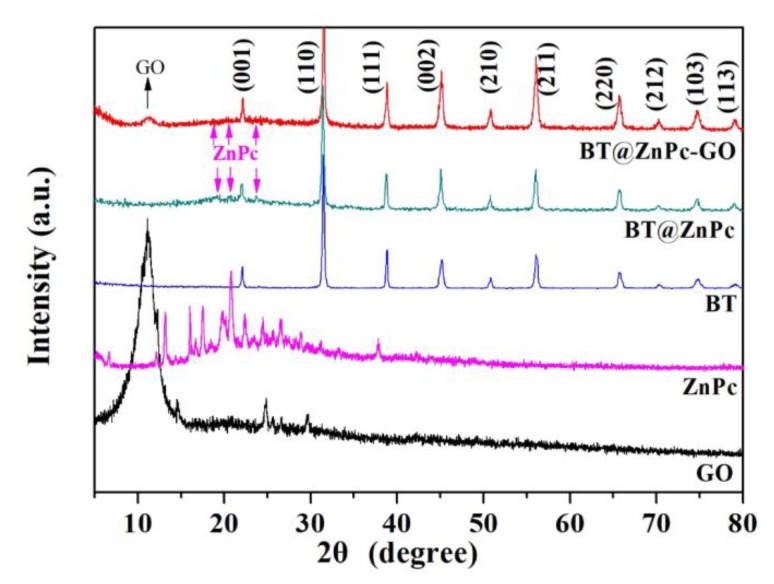
X-ray diffraction (XRD) patterns of GO, ZnPc, BT, BT@ZnPc and BT@ZnPc-GO.

Furthermore, the the XRD patterns of GO, ZnPc, BT, BT@ZnPc and BT@ZnPc-GO are shown in Figure 5. As can be seen from the spectra of BT, BT@ZnPc and BT@ZnPc-GO that the diffraction patterns at 2θ = 22.2°, 31.4°, 38.5°, 45.2°, 50.7°, 56.1°, 65.6°, 74.7° and 79.5° which responding to the (001), (110), (111), (002), (210), (211), (001), (220), (212), (103) and (113), showing the crystal planes of BT exhibiting the cubic phase [26]. Meanwhile, ZnPc exhibits halo peaks from 10°–40° [24]. From the spectra of BT@ZnPc and BT@ZnPc-GO in the figure, it can be found that they have the same diffraction peaks at 10°–40°, which proves the existence of ZnPc in the BT@ZnPc and BT@ZnPc-GO nanoparticles. It is worth mentioning that the same diffraction peak as graphene oxide appears in the BT@ZnPc-GO spectrum, which indicates that graphene oxide has been successfully modified in BT@ZnPc-GO.

All these results prove that BT@ZnPc-GO nanoparticles have been successfully prepared in this work.

**Figure 6:**
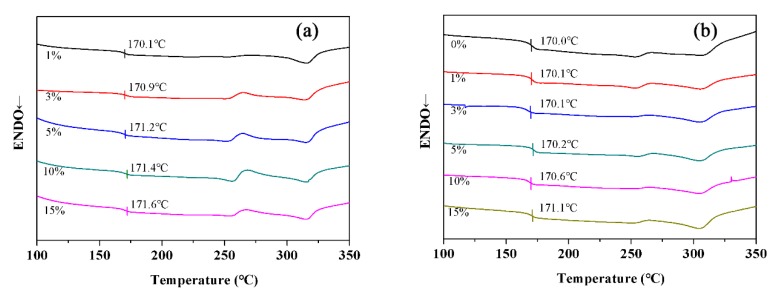
DSC curves of (**a**) BT@ZnPc-GO-1/PEN films with 1, 3, 5, 10 and 15 wt % of BT@ZnPc-GO-1 and (**b**) BT@ZnPc-O-2/PEN films with 1, 3, 5, 10 and 15 wt % of BT@ZnPc-GO-2.

Based on the successful preparation and characterization of the BT@ZnPc-GO nanoparticles, they were incorporated into the PEN matrix to prepare the BT@ZnPc-GO/PEN composite films by the solution-casting method, and the thermal and dielectric properties were investigated in detail. Figure 6 shows the DSC curves of the BT@ZnPc-GO/PEN nanocomposites. As can be seen from the figure that the glass transition temperature (*T*_g_) of the pure PEN was about 170.0 °C, and with the increase of the BT@ZnPc-GO content, the *T*_g_ of the BT@ZnPc-GO/PEN nanocomposites present a slight increase. This is because the nanoparticles are regarded as the physical crosslink points in the PEN matrix, which limit the movement of molecular chains, resulting in improvement of the *T*_g_ of the nanocomposites [27]. In addition, the PEN used herein has a certain degree of crystallization, which is due to the large number of rigid p-phenyl and m-benzene segments contained in this structure (Scheme 1). More importantly, double melting points are obviously seen from the DSC spectrum of the all samples in Figure 6. This is mainly caused by the copolymer of HQ-PEN block and RS-PEN block forms two different crystals, which has been reported in previous literature by Wang, et al. in our group [28].

Furthermore, the crystallization of the BT@ZnPc-GO/PEN nanocomposites shows no obviously changes with the increase of the BT@ZnPc-GO content.

**Figure 7:**
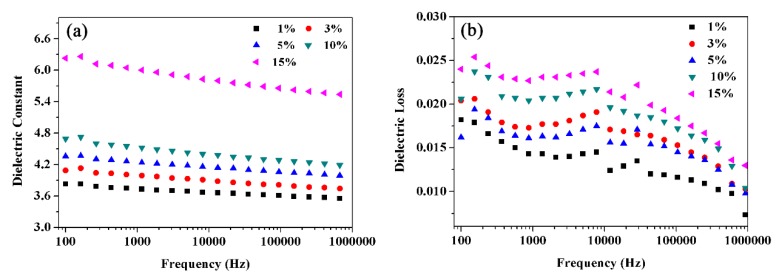
Dielectric constant (**a**) and dieledctric loss (**b**) of PEN composites with 1, 3, 5, 10 and 15 wt % of BT@ZnPc-GO-1.

For research the dielectric behavior of BT@ZnPc-GO/PEN nanocomposites with different BT@ZnPc-GO contents, the dielectric properties of nanocomposites were measured as a function of frequency from 100 Hz to 1 MHz. The dielectric constant and loss of the BT@ZnPc-GO-1/PEN composites is shown in Figure 7. It is obvious that the dielectric constant of the nanocomposites presents a gradual upward trend (from 3.75 to 6.05 at 1 kHz) as the increment of the BT@ZnPc-GO-1 (from 1% to 15 %), which is shown in Figure 7a. This is mainly owing to that the nanoparticles in the PEN matrix can be regarded as forming the micro-capacitors networks. With the increase of nanofiller content, the number of micro-capacitors increases, resulting in increasing the dielectric constant of nanocomposites. In addition, it worth noting that when the BT@ZnPc-GO-1 nanoparticles are added to 15 wt % this indicates the amount of graphene is only 7.5 wt ‰, and the dielectric constant of the nanocomposites is 6.2. According to previous reports [20], when the amount of ZnPc@BT is added as high as 30%, the dielectric constant of the composites is still about 6.0. This reveals that a significant increase in dielectric constant is achieved with low additions of graphene, which is due to the fact that heterojunction between the conductor and the ferroelectric increases the dielectric constant of the composites [29]. Meanwhile, the mismatch in conductivity between the two phases of GO and ZnPc results in more and more free carriers accumulating at the interface and producing strong interfacial polarization, which further leads to an increase in dielectric constant. Furthermore, it is not hard to find that the dielectric constant of all nanocomposites decreases slightly with the increase of frequency, which is due to the polarization relaxation process [30]. However, when the content of BT@ZnPc-GO-1 nanoparticles was 15%, the change rate of dielectric constant was only 10.9% in the range of 100 Hz to 1 MHz, which suggested that the nanocomposites have a good dielectric constant-frequency stability. This indicated that these nanocomposites can be used as the dielectric materials in the field of electronic components.

At the same time, the dielectric loss of the nanocomposites shows a similar trend to that of the dielectric constant in this work. It can be seen from Figure 7b that the dielectric loss slightly increased from 0.018 to 0.023 at 1 kHz with the BT@ZnPc-GO-1 contents increasing from 1% to 15%, which is mainly caused by the conductance loss [31]. More importantly, the dielectric loss of all nanocomposites was well controlled at a relatively low level (<0.025), which satisfied the practical application requirements of electronic components.

**Figure 8:**
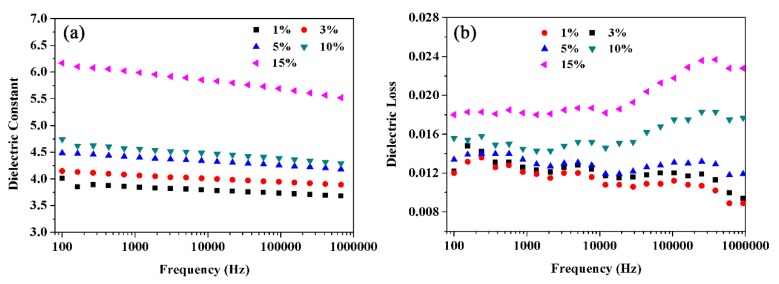
Dielectric constant (**a**) and dieledctric loss (**b**) of PEN composites with 1, 3, 5, 10 and 15 wt % of BT@ZnPc-GO-2.

In addition, compared with the dielectric properties of the BT@ZnPc-GO-1/PEN nanocomposite films, the dielectric constants of the BT@ZnPc-GO-2/PEN nanocomposite films showed the same change trend to that of BT@ZnPc-GO-2/PEN, which are shown in Figure 8a. Besides, it is clear that the dielectric constant of the BT@ZnPc-GO-2/PEN was slightly lower than that of BT@ZnPc-GO-1/PEN at the same content of the nanofillers, which was due to the increase in graphene oxide content. In addition, the dielectric loss of the nanocomposites tends to increase at high frequencies.

Therefore, according to the dielectric properties of the nanocomposite films, the BT@ZnPc-GO-1/PEN nanocomposites with 15 wt % BT@ZnPc-GO-1 present a relatively excellent dielectric property, which is selected to study the effect of the crystallization behavior of the nanocomposite films on its dielectric properties.

**Figure 9:**
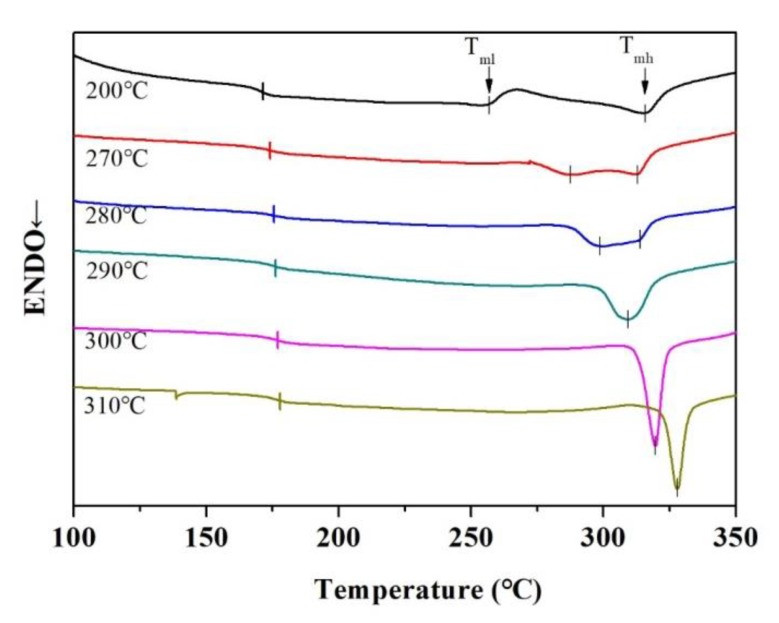
DSC curves of BT@ZnPc-GO-1/PEN films with 15 wt % BT@ZnPc-GO-1 after isothermal annealing at 200, 270, 280, 290, 300 and 310 °C for 2 h.

In order to further investigate the crystallization behavior of the nanocomposite films, the BT@ZnPc-GO-1/PEN nanocomposites were annealed at different temperatures (200 °C to 310 °C) for 2 h. The annealed films were cooled to room temperature prior to DSC measurementand all the data related to crystallization properties are summarized on Table 1. Figure 9 shows the DSC curves of nanocomposites after isothermal crystallization for 2 h at different temperatures. As can be seen from the figure, the BT@ZnPc-GO-1/PEN nanocomposites have two melting points (where *T*_ml_ is 256.35 °C, *T*_mh_ was about 314.51 °C). It is interesting that as the isothermal treatment temperature increases, *T*_mh_ remains constant and the *T*_ml_ shows a gradual increasing trend. This is obviously being caused by the newly formed crystalline regions being affected during the heat-treatment process. In addition, the arrangement of macromolecules in the amorphous phase can also result in thickening of the original crystalline regions. However, as the processing temperature increases, more and more macromolecules in the amorphous phase rearrange to form new crystalline regions, resulting in an increase in new crystalline regions and an increase in *T*_ml_ [31]. As shown in Figure 9, when the heat treatment temperature is further increased and approaches *T*_ml_, the macromolecules in the original crystalline region start to move, resulting in disintegration of the crystalline region. The disintegration of the crystalline region leads to a decrease in melting enthalpy. In addition, the BT@ZnPc-GO-1/PEN nanocomposites isothermal treatment at 280 °C for 2 h show the highest melting enthalpy (=15.33 J), which indicated that 280 °C is the best isothermal crystallization temperature in this system. Furthermore, another phenomenon than can clearly be seen in Figure 9 is that as the isothermal processing temperature increases, the *T*_g_ of the nanocomposites increases gradually. This is because the crystal limits the motion of the macromolecular backbone of the sample [28].

**Figure 10:**
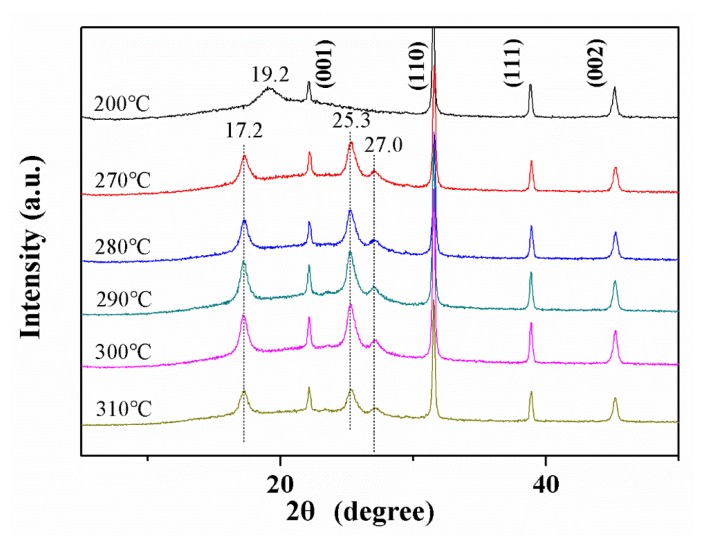
XRD patterns of BT@ZnPc-GO-1/PEN nanocomposite films with 15 wt % BT@ZnPc-GO-1 after isothermal annealing at 200, 270, 280, 290, 300 and 310 °C for 2 h.

Figure 10 shows the XRD diffraction spectrum of the BT@ZnPc-GO-1/PEN film treated at different temperatures, which further characterized the crystallization behavior of the nanocomposites. As can be seen from the figure, the diffraction angle remaining after removing the diffraction angle of BT is the diffraction angle of the PEN-based nanocomposite films. In addition, the nanocomposites treated at 200 °C show a only diffraction peak at 19.16°. According to previous reports on the crystallization behavior of this structured PEN [31], the PEN based composite films cannot crystallize well by being treated at 200 °C, resulting in the formation of many incomplete crystals. When the composite film is processed at a higher temperature, the film shows three diffraction peaks at 17.25, 25.3 and 27.0°, which is many caused by these factors: on the one hand, some incomplete crystals have been recrystallized in the process of high-temperature isotherm treatment, forming the new complete crystals; on the other hand, some molecular chains of the PEN in the amorphous phases are rearranged at high temperatures, and gradually form a new crystalline phases [28]. When the isothermal treatment temperature is raised from 270 °C to 280 °C, the crystallinity of nanocomposites shows an increasing tendency, from 18.20% to 21.03%. This result indicates that an increase in temperature favors the formation of a crystal structure. However, as the temperature is further increased to 310 °C, the crystallinity of the nanocomposites will gradually decrease from 21.03% to 17.56%, which may be due to the fact that once the temperature exceeds 280 °C, the molecular chain in the crystal phase will begin to move, destroying the crystal structure. The same phenomenon is consistent with that observed from the DSC results. Moreover, with the addition of a nucleating agent in the PEN matrix, the nucleating agent promotes crystallization while also reducing the optimal crystallization temperature.

**Figure 11:**
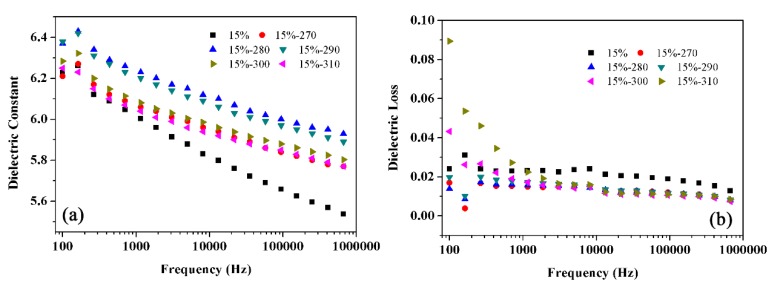
Dielectric constant (**a**) and dielectric loss (**b**) of BT@ZnPc-GO-1/PEN films after isothermal annealing at 200, 270, 280, 290, 300 and 310 °C for 2 h.

It has been reported that the crystallization behavior of the polymer will affect the dielectric properties of the polymer based composites [27]. Figure 11 shows the dielectric properties of BT@ZnPc-GO-1/PEN composite film as a function of heat-treatment temperature. It can be seen from the figure that the heat-treatment conditions have an effect on the dielectric properties of the BT@ZnPc-GO-1/PEN nanocomposites. When the heat-treatment temperature is raised from 200 °C to 280 °C, the dielectric constant of the nanocomposites increases from 6.00 to 6.24 at 1 kHz. This is due to the fact that the nanofillers are mainly dispersed in the amorphous phase of the polymer matrix, and when the degree of crystallization increases, the fillers will be more dense in the amorphous phase [28]. From the perspective of percolation theory, the concentration equivalent to BT@ZnPc-GO-1 is improved, which will improve the dielectric constant of the nanocomposites [32]. From the perspective of microcapacitor theory, although the total number of microcapacitors is the same, the pitch of the capacitor plates is small, and the capacitance of a single capacitor increases, resulting in improving and contributing to the dielectric constant of the nanocomposites [33]. Nevertheless, once the heat-treatment temperature continuously increases, the dielectric constant of the nanocomposite will also decrease causing decreasing crystallinity. However, the dielectric constant of nanocomposites after heat treating at 310 °C is still higher than that treated at 200 °C. More importantly, the dielectric loss of nanocomposites by heat treatment at different high temperatures is quite lower than that treated at 200 °C. All these results indicate that the isothermal crystallization behavior has certain positive significance for improving dielectric properties.

**Figure 12:**
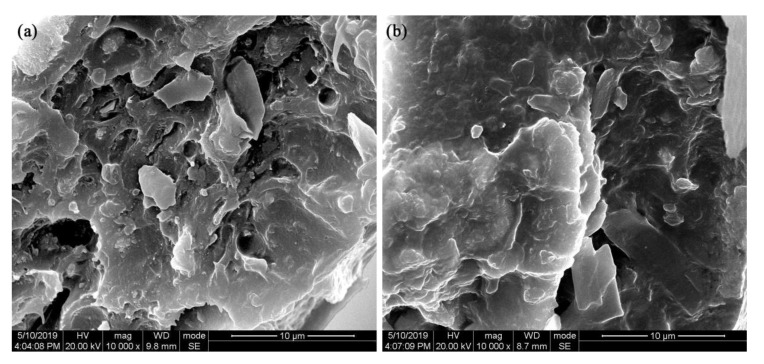
SEM images of the BT@ZnPc-GO-1/PEN films with 15 wt % BT@ZnPc-GO-1 nanoparticles (**a**) before and (**b**) after isothermaol annealing at 280 °C for 2 h.

In order to explore the compatibility of BT@ZnPc-GO nanoparticles with the PEN matrix, the cross-section morphology of the nanocomposites is further investigated by SEM measurement. Figure 12 shows the SEM micro-images of the BT@ZnPc-GO-1/PEN films with 15 wt % BT@ZnPc-GO-1 nanoparticles before and after isothermal annealing at 280 °C for 2 h. As can be seen from the figure, the BT@ZnPc-GO nanoparticles have a good compatibility with the PEN matrix, and there is no obvious phase interface. In addition, all the nanoparticles are evenly dispersed in the PEN matrix without obvious agglomeration and the content of nanofillers is 15 wt %. This result is mainly due to the fact that the surface functionalized BT nanoparticles are uniformly attached onto the surface of GO nanoparticles to form a new relatively stable hybrid, which effectively promotes its dispersion in PEN matrix. Meanwhile, the organic zinc phthalocyanine-functionalized BT nanoparticles can effectively promote the compatibility of BT and PEN matrix [20]. All these results indicate that a new method for preparing high-performance PEN-based nanocomposites has been established to fabricate film-capacitor assemblies for use in high temperature environments.

## 4. Conclusions

In summary, in this work barium titanate@zinc phthalocyanine/graphene oxide (BT@ZnPc-GO) nanoparticles with different GO contents were successful prepared by electrostatic adsorption between BT@ZnPc and GO nanoparticles with the action of calcium ions, and the BT@ZnPC-GO nanoparticles could be adjusted and controlled by adding different amounts of GO. The obtained nanoparticles were characterized in detail by TEM, XPS, XRD and FTIR to confirm that the BT@ZnPc nanoparticles were deposited on the graphene nanosheet surface. Thereafter, two kinds of nanoparticles were combined in a compound with a PEN matrix to prepare the BT@ZnPc-GO/PEN nanocomposites. The results present that the obtained BT@ZnPc-GO nanoparticles show good compatibility with PEN. In addition, with the BT@ZnPc-GO content increased from 0 to 15 wt %, the dielectric constant of the BT@ZnPc-GO/PEN nanocomposites increased from 3.6 to 6.2, and the dielectric loss was still less than 0.25. Furthermore, the nanocomposites also exhibited excellent thermal stability (*T*_g_ > 170 °C). More importantly, the BT@ZnPc-GO nanoparticles can be used as nucleating agent to promote the crystallization of the nanocomposites from 13.21% to 21.03% during the isotherm heat-treatment process, which will further improve the dielectric properties of the nanocomposites. All these results indicate that a new method for preparing high-performance PEN-based nanocomposites has been established to fabricate film-capacitor assemblies for use in high-temperature environments.

## Figures and Tables

**Table 1:** The physical parameter values of BT@ZnPc-GO-1/PEN films with 15 wt % BT@ZnPc-GO-1 after isothermal annealing at 200, 270, 280, 290, 300 and 310 °C for 2 h.

Tempreture (°C)	200	270	280	290	300	310
Melting enthalpy ΔH (J g^−1^)	9.63	13.27	15.33	14.74	13.79	12.80
Melting point (*T*_ml_, °C)	257.38	287.35	298.63	309.33	319.89	328.26
Melting point (*T*_mh_, °C)	316.22	313.02	314.29	309.33	319.89	328.26
Glass transition temperature (°C)	171.36	173.62	175.67	176.24	177.01	178.08
Crystallinity (%)	13.21	18.20	21.03	20.22	18.92	17.56
